# Development of a Conductive Polymer Based Novel 1-DOF Tactile Sensor with Cylindrical Arch Spring Structure Using 3D Printing Technology †

**DOI:** 10.3390/s19020318

**Published:** 2019-01-14

**Authors:** Peshan Sampath, Eranga De Silva, Lakshitha Sameera, Isuru Udayanga, Ranjith Amarasinghe, Sampath Weragoda, Atsushi Mitani

**Affiliations:** 1 Department of Mechanical Engineering, University of Moratuwa, Katubedda 10400, Sri Lanka; ahteranga@gmail.com (E.D.S.); nupehewagels@gmail.com (L.S.); ranama@uom.lk (R.A.); 2 Department of Engineering Technology, University of Sri Jayewardenepura, Gangodawila, Nugegoda 10250, Sri Lanka; isurutd@sjp.ac.lk; 3 Department of Materials Science and Engineering, University of Moratuwa, Katubedda 10400, Sri Lanka; sampathw@uom.lk; 4 Department of Design, Sapporo City University, Hokkaido 005-0864, Japan; a.mitani@scu.ac.jp

**Keywords:** sensor phenomena and characterization, sensor structures, springs, tactile sensors, three-dimensional displays, 3D printing

## Abstract

Under this research, a novel tactile sensor has been developed using a conductive polymer-based sensing element. The incorporated sensing element is manufactured by polymer press moulding, where the compound is based on silicone rubber and has enhancements by silica and carbon black, with Silane-69 as the coupling agent. Characteristics of the sensing element have been observed using its sensitivity and range, where its results pose an inherent nonlinearity of conductive polymers. For the force scaling purpose, a novel 3D printed cylindrical arch spring structure was developed for this highly customizable tactile sensor by adopting commonly available ABSplus material in 3D printing technology. By considering critical dimensions of the structure, finite element analysis was carried out to achieve nearly optimized results. A special electrical routing arrangement was also designed to reduce the routing complexities. The optimized structure was fabricated using the 3D printing technology. A microcontroller-based signal conditioning circuit was introduced to the system for the purpose of acquiring data. The sensor has been tested up to the maximum load condition using a force indenter. This sensor has a maximum applicable range of 90 N with a maximum structural deflection of 4 mm. The sensor assembly weighs 155 g and the outer dimensions are 85 mm in diameter and 83 mm in height.

## 1. Introduction

Tactile sensors are capable of capturing electronic sensing signals using tactile sensing principles and measuring tactile parameters with the assistance of physical touch. Tactile parameters may often include temperature, vibration, softness, texture, shape, shear and normal force [1]. Even though pressure and torque are not identified as tactile parameters in this list, they are important parameters that can be sensed through physical touch [1,2,3]. Even though tactile sensing came to the attention of researchers in the 1970s, tactile sensors have not been developed much compared to the non-contact sensors [4,5], due to the direct physical contact forces/impacts that have to be manipulated by the sensors/sensor-structures themselves. With the recent advances in the robotics and automation field, there is a requirement for tactile sensing feedback systems that performs better with respect to force range, dynamic range, frequency response and special resolution [6]. Henceforth, a large amount of research has been carried out on the subject of tactile sensing and numerous devices have been developed for different applications [2,7,8]. In addition, application areas of tactile sensing have been expanded as new fields of applications have emerged in recent times.

Although human tactile sensing or, more specifically, human skin provided a reference point to researchers when developing tactile force sensors, tactile sensors are only capable of sensing a lesser number of tactile parameters simultaneously [9]. Current tactile force sensors face difficulty in meeting the demands of force sensors required in modern sophisticated measurement systems and control systems.

During the recent decades, many sensing principles have been identified that can be associated with tactile force sensors, such as piezo-electric [10], optical [11], piezo-resistive [12,13], conductive polymer/composite [14] and so on. Each of these sensing principles has their own advantages and disadvantages [1,3]. Hence, the use of them in applications should be considered according to the performance required. Conductive rubber based piezoresistive force sensors have the benefits of good sensitivity, low noise, simplicity in electronics, low-cost manufacturing and physical flexibility of the sensing element. If the conductive rubber based piezoresistive force sensing element equips the intra-electron tunnelling effect, other than the classical percolation piezoresistivity, the sensitivity would be further enhanced. As for drawbacks, conductive rubber based piezoresistive force sensors are behaving nonlinearly in response to hysteresis, signal drift and temperature sensitivity [9]. Henceforth, conductive rubber based piezoresistive force sensors have to be used in a steady temperature environment with thorough signal conditioning.

Robotics, entertainment, medical and safety systems fall under a few of those application domains of pressure/force sensors [2]. Moreover, force sensors could be used for overloading detection systems, robotic manipulator feedback [2], weight measurements, prosthetic/rehabilitation devices [7] and impact testers [8]. Contemporary tactile force sensor research has focussed on areas such as usage of flexible materials [15], simplified fabrication using rapid prototyping techniques [16,17] and sophistication of the sensing element [18]. With the inherent capabilities of 3D printing, the limitations of planar microfabrication processes can also be overcome in sensor designing. Hence, 3D printed tactile sensors can be easily integrated or fitted into any complex space with convenient packaging and convenient electrical routing, even with peripheral force scaling structures.

Tactile sensors have been developed mainly with serial mechanisms [19] and parallel mechanisms [20]. Further designs have been done with cross beam structures [21,22], deflecting bar structures [23], T-shaped bar structures [24] and force scaling support structures [13]. On the other hand, tactile switches with novel structures are proliferating due to the demand in consumer electronics [25]. Nevertheless, state-of-the-art propensity of tactile sensor design is towards 3D printed tactile sensors [26], whereas novel complex shapes could be conveniently fabricated without any hurdle according to the needs of the sensor researchers.

Even though sensorised soft structure designs are included among contemporary research [26,27], many drawbacks exist. Poor adhesion (non-compatibility) between the soft sensing material and the sensing material is an issue [28], where the selection of material is limited to those with sufficient adhesion (otherwise, the integrity is lost). Structural analysis complexity, where sudden spatial transition in material stiffness causes high stress concentrations [28] and high cost of sensor inks [29] are among the other drawbacks in sensorised soft structures over tactile sensors with 3D printed force scaling/supported structures.

The force scaling structure which was designed and developed in this study is novel and unique with respect to the state-of-the-art sensor structures. Thereupon, this novel sensor structure offers a greater customisability in design and further enhancing compactness.

In this study, a unique structure has been developed for a tactile sensor with a conductive polymer based sensing element. The sensing structure was built based on cylindrical arch shaped springs used as base units which are there to accomplish required mechanical characteristics. Output signal conditioning was carried out using the electrical wiring system incorporated to the sensor structure. The proposed design for the structure was validated before the fabrication process, which used 3D printing technology.

Due to the ease of customisability, the applications of 3D printed force sensors have a large scope in fields such as gait analysis floor sensor systems [30], anti-theft security systems, adaptive vehicle ergonomics [31], human motion tracking systems [32], consumer electronic interfacing devices [25] and fuzzy logic force feedback systems [33].

## 2. Elements of the Sensor and the Working Principle

**Figure 1:**
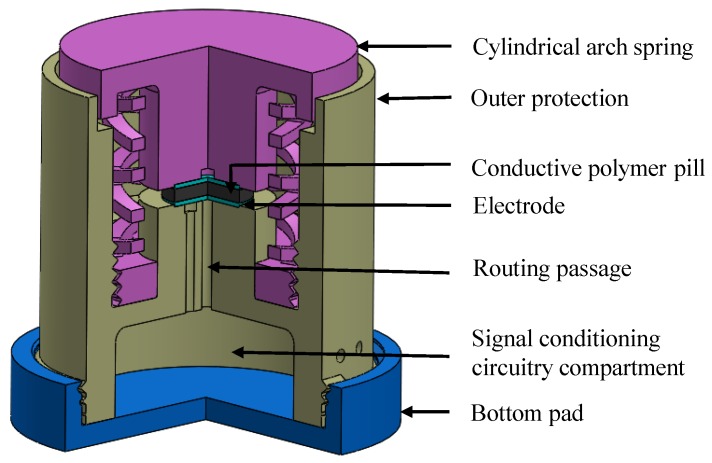
A three-quarter view section of the proposed force sensor design.

**Figure 2:**
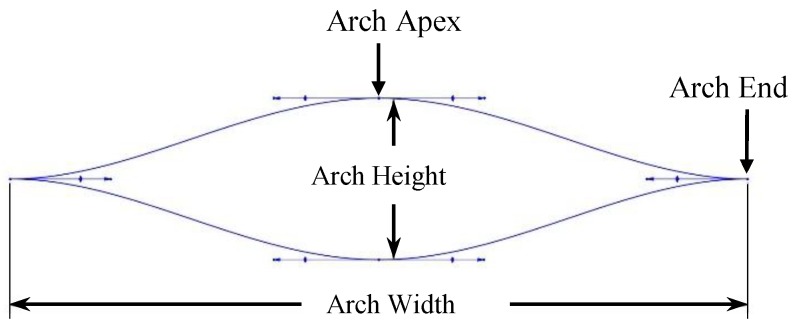
A single cylindrical arch design and the arch aspect ratio.

When considering a force sensor, it is very important to restrain the total deflection of the sensing element so that the desired range of the force sensor can be achieved. One method to restrain the total deflection is to introduce an exterior spring system. An external cylindrical spring system (as shown in Figure 1) was introduced for the proposed structure of the sensor to fulfill the above requirement. Furthermore, the alterations in the arch spring arrangement and dimensions could enhance the output characteristics of the sensor. A three-points spline which has zero gradients at the apex and ends was adopted for a single cylindrical arch design (as shown in Figure 2) to obtain the deformation that is necessary for the piezo-resistive behaviour.

**Figure 3:**
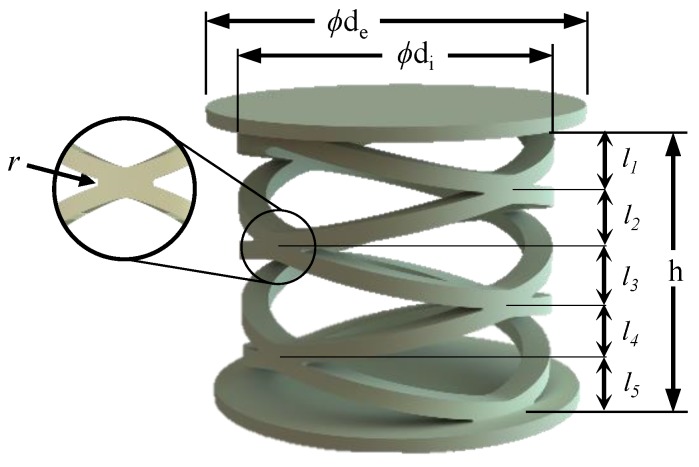
Cylindrical arch spring structure.

External diameter of spring system ($\phi d_{i}$), diameter of support layer ($\phi d_{e}$), total spring height (*h*) and fillet radius (*r*) shown under Figure 3 were considered as major design dimensions of the proposed arch spring arrangement.

The force sensor structure was also designed inclusive of an overload protection to the structure. The vertical guide of the cylindrical arch spring is 3D printed with a maximum gap of 4 mm to serve as overload protection for normal forces. A shear force that can be applied on the force acting platform of the structure would be mitigated by the clearance of 200 μm kept between the linear guide and the outer protection.

## 3. Design and Development of the Tactile Sensor

### 3.1. Cylindrical Arch Spring Structure

#### 3.1.1. Development of Cylindrical Arch Spring Structure

The extent of the piezo-resistive elements described under Table 1 and the arrangement limitations of the universal testing equipment were taken into account when designing the cylindrical arch spring system shown in Figure 3. Later, the design was developed and carefully analysed with the help of COMSOL Multiphysics using its Finite Element Analysis tools.

During the Finite Element Analysis (FEA), the sensing pill inside the structure was modeled and simulated as a virtual spring. Then, the combined system was analysed using FEA tools by applying a prescribed displacement of 2.5 mm for the sensing element (virtual spring) along the center axis. It was inspected during the static load test that the sensing elements exhibit a maximum of 2.5 mm displacement at the expansion of its range. Analysed maximum von-Mises stress, ($3.42\times 10^{7}$ N/m^2^) was well within the maximum tensile strength of ABSplus ($3.6\times 10^{7}$ N/m^2^) [34], which was used for the 3D printing fabrication process.

**Figure 4:**
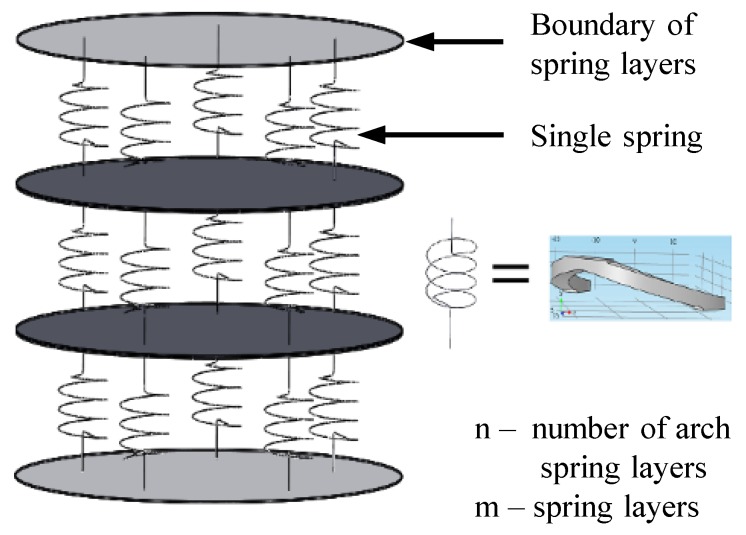
Cylindrical arch spring structure was analytically modelled for further modifications.

The arch spring structure was simplified to a parallel and series spring system and used to introduce further modifications to the spring structure. The simplified analytical model is shown in the Figure 4 and it is equivalent to the proposed design seen in Figure 1. The number of arch spring layers are represented as “n” (which have been depicted as $l_{1}$, $l_{2}$, …‥ and $l_{5}$ in Figure 3 for the proposed structure). The number of parallel springs between two boundaries (shown in Figure 4) of the spring layer are indicated as “m”. Equation (1) would give the representation for the axial spring constant of the spring structure:

(1) $$k_{s}=m\times \frac{k_{a}}{n},$$

where the stiffness of the arch spring structure and the stiffness of a single arch are represented by $k_{s}$ and $k_{a}$, respectively.

**Figure 5:**
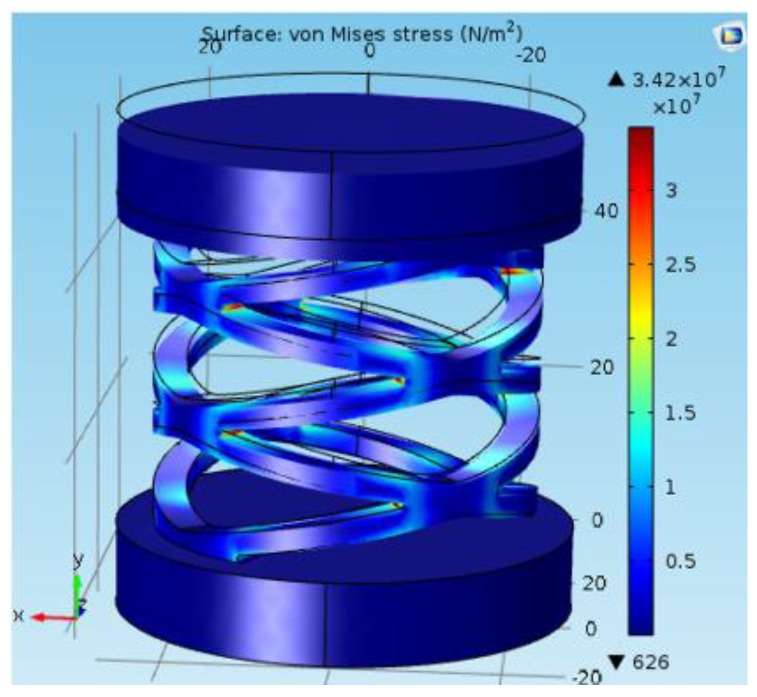
Structural simulation of arch spring structure.

**Figure 6:**
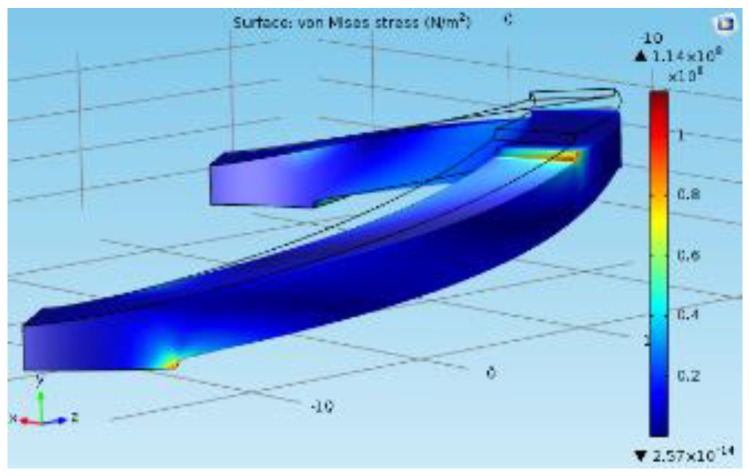
FEA study for a single cylindrical arch where the apex boundary is subjected to a force component.

The FEA study was carried out using the COMSOL Multiphysics 5.0 to justify the analytic model for the same model used for the structural simulation (shown in Figure 5). In the analysis conducted for the single cylindrical arch spring Figure 6, a force component is given to the topmost surface of the spring in order to calculate the maximum deformation. Stiffness for a single cylindrical arch and the entire spring structure (without the consequence of the sensing element) were derived as 25,673 N/m and 10,888 N/m, respectively. Equation (1) could be verified by the above results with an error percentage of 6.02%. whose causes could be the assumptions of the equations and the round-off errors in the FEA study.

In order to determine the necessary stiffness of the single cylindrical arch spring portion which is to be implemented for Force Range (FR) increment per given deflection, Equation (2) could be used, which was derived via the basic equation for spring stiffness:

(2) $$FR_{extended}=\frac{k_{p}+\frac{m}{n}k_{a}}{k_{p}}\times FR_{sensor\;pill}$$

where $k_{p}$ is stiffness of the sensor pill which is mounted according to Figure 1. The nonlinearity of the sensing element and the spring structure should have to be eliminated at the manufacturing stage by enhancing the material composition of the sensor pill and by adjusting the 3D printing parameters, respectively.

**Figure 7:**
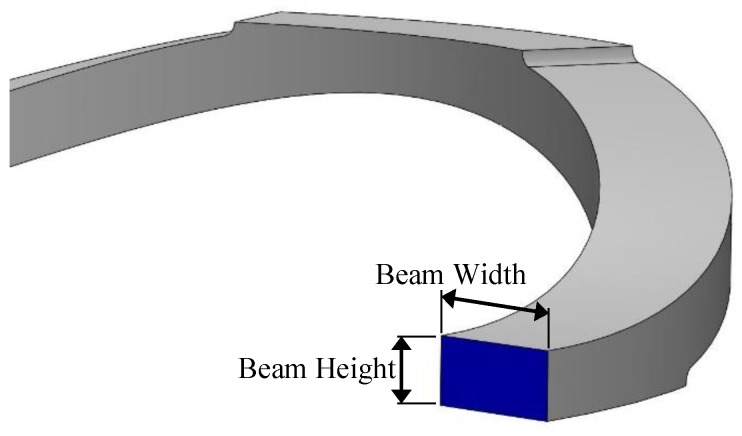
Beam aspect ratio.

The arch spring structure was prototyped according to the failsafe verifications that were obtained from the FEA study, where the prototyped sensor had five layers of arch springs and two arch springs in parallel in each layer. In order to attain a desired force sensor range, the prime aspect would be to design a single arch with a specific stiffness by considering its width, height (shown in Figure 2), beam thickness and height (shown in Figure 7). In each arc, beam width, beam height, arch width and arch height are 4 mm, 2 mm, 55 mm and 12 mm, respectively, following the terms that were referenced in Figure 2 and Figure 7.

**Figure 8:**
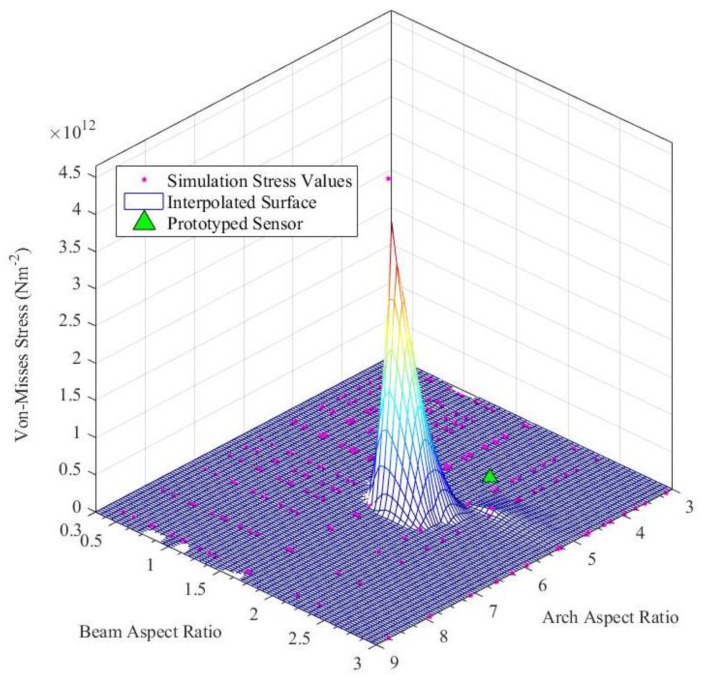
Von-Mises stress vs. structural design parameter study for the cylindrical arch spring.

**Figure 9:**
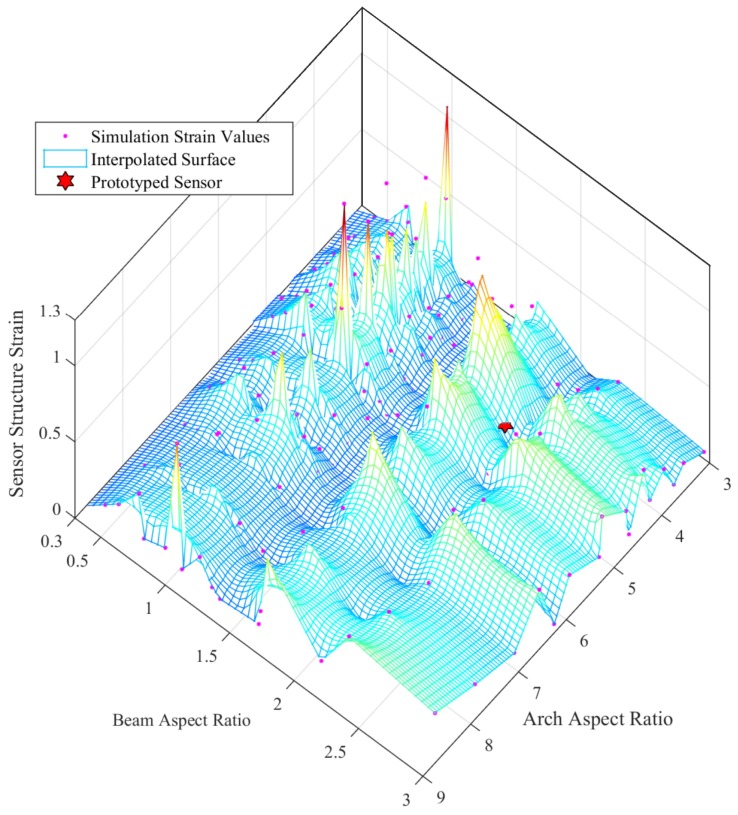
Sensor strain vs. structural design parameter study for the cylindrical arch spring.

Considering the Arch Aspect Ratio (=arch width/arch height) and the Beam Aspect Ratio (=beam width/beam height), which are two major design parameters, a parametric study was conducted with adaptive mesh refinements for each parametric step. Figure 8 and Figure 9 depicts the results of the parametric study and status of the prototyped cylindrical arch spring structure. Hence, this parametric design methodology (the results of the parametric study) could be adhered to by a sensor designer in order to obtain the desired characteristics out of a sensor by altering the structural parameters. Dimensions of the sensor packaging and application specific constraints must also be considered. Despite the validated single arch spring, the entire cylindrical arch spring structure should be thoroughly validated by simulations in order to be fail-safe prior to the fabrication, as the arch crevices are the areas with the highest stress concentration.

#### 3.1.2. Fabrication of the Sensing Structure

ABSplus production-grade thermoplastic is a material for prototyping through direct digital manufacturing, which possess 36.0 MPa of tensile strength. ABSplus can 3D print components directly from digital files which are stronger and smoother in finish—hence with more feature details. Moreover, ABSplus has a higher adhesion between 3D printed layers [35]. In practice, ABSplus is known for its resilience to warping after fabrication, where the misalignments and assembly misfits could be minimised.

When considering the tensile moduli of 3D printing materials, ABSplus [34], ABS [36] and PLA [37] possess tensile moduli of 2.265 GPa, 2.3 GPa and 2.76 GPa, respectively. The failure stresses of the ABSplus, ABS and PLA are 36.0 MPa, 22.0 MPa and 26 MPa, respectively. Thereupon, ABSplus was selected as it possesses the minimum failure stress to tensile modulus index value, since the sensor structure manipulates the stiffness by the geometry and the strength should be safeguarded by the material itself.

**Figure 10:**
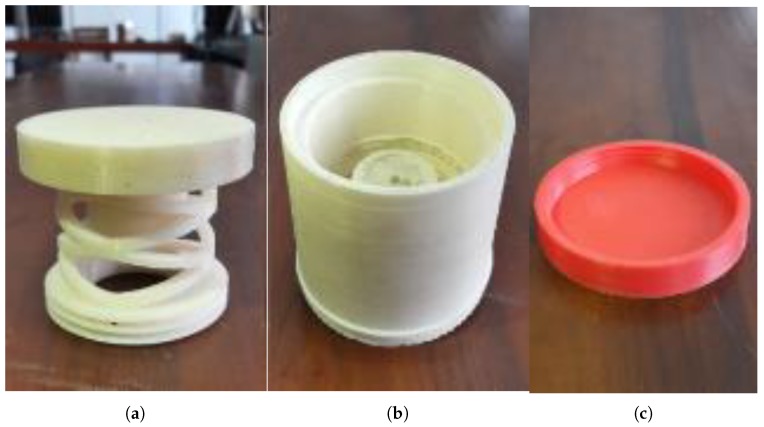
3D printed parts of the force sensor: (**a**) cylindrical arch spring; (**b**) outer protection; (**c**) bottom pad.

ABSplus [34] used with 3D printing technology was used to fabricate structural parts of the sensor (Figure 10). The outer protection (Figure 10b) and the bottom pad (Figure 10c) are fabricated using the Dreamer Flashforge 3D printer. The other part(Figure 10a) is fabricated using the Stratasys Uprint SE 3D printer with an infill ration of 100% and a layer thickness value of 0.254 mm as it is vital to bear high stresses as much as $3.42\times 10^{7}$ N/m^2^.

### 3.2. The Sensing Element

The sensing element used in this paper was developed using conductive polymers that have received vital consideration in the fields of science and engineering because of its allure in various range of electrical conductivity, which can be obtained with various doping levels while sustaining its mechanical flexibility and high thermal stability. Such materials possess piezo resistivity that is capable of changing its conductive properties by changing its geometry. The range of the piezo-resistive element is highly dependent on the sensing material composition and the geometry of the element.

#### 3.2.1. Development of the Sensing Element

The developed conductive polymer based sensing element is mainly based on Room Temperature Vulcanization (RTV) Silicone Rubber and mixed with the addition of Nano-materials (super conductive carbon black and nano silica ($SiO_{2}$)) to enhance its performance using a shear mixing process adopting the method described by Huang et al. [38].

The manufacturing method for the optimised conductive rubber is such that, at normal room temperature and pressure, the ECP-CB-1 high conductive carbon black and Si-69 silane coupling agent are mixed together. Nano SiO_2_ is then added to the mixture. Finally, single-component RTV Silicone rubber is added to the mixture using a shear-kneading machine. The sensing elements should be made by injecting the mixed solution to a press mould with a solidification time of 64 to 72 h.

Table 1 shows the dimensions of the sensing element that was produced. It is capable of varying the maximum applicable load by changing the pill thickness. The sensing element incorporated into the sensing structure which was used for the experiments of this study had a diameter (D) and a height (H) of 20 mm and 10 mm respectively, as illustrated in Figure 1.

#### 3.2.2. Fabrication of the Sensing Element

**Figure 11:**
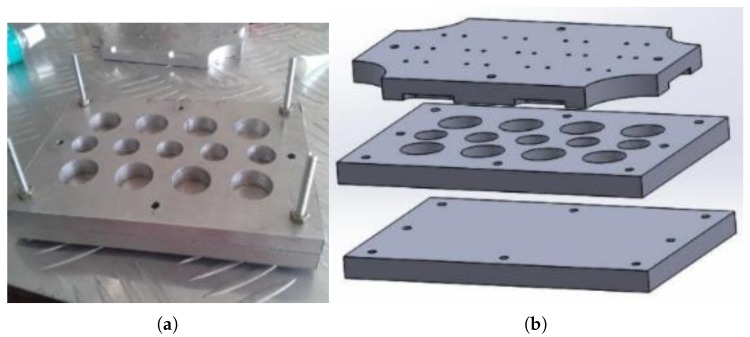
Mould used to fabricate sensor pills: (**a**) aluminium—press mould; (**b**) exploded view of the mould.

**Figure 12:**
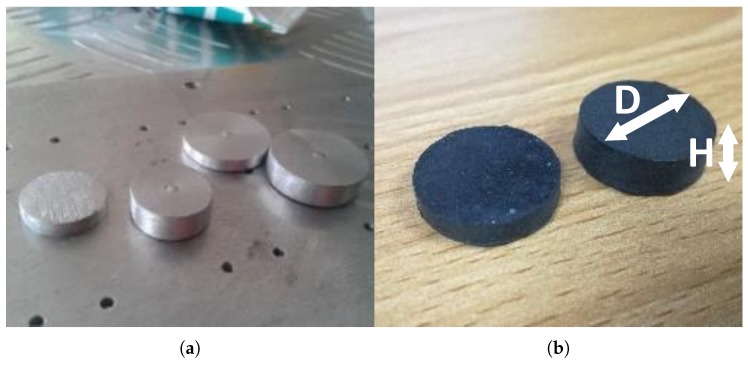
Fabrication of the sensor pills: (**a**) spacers to alter the thickness values of the pills; (**b**) conductive polymer pills after the fabrication.

Fabricated conductive polymer based sensing material was developed based on Silicone Rubber and can be customised to any required dimension according to the mould (Figure 11) used. The thickness of the sensing element pills could be altered by the spacers that could be inserted into the mould as shown in Figure 12a to make conductive polymer pills with different geometries as shown in Figure 12b. A mechanical press moulding method is used to develop the sensing element while the mould described under Figure 11 is designed into three layers to ease the ejection process of the sensing element from the mould.

The capability of the mould to develop sensing elements with varying geometries and thicknesses in a single batch of production is a key feature of this production process as the sensing element can be developed according to the required geometry and size with different performance levels.

### 3.3. Electrodes

**Figure 13:**
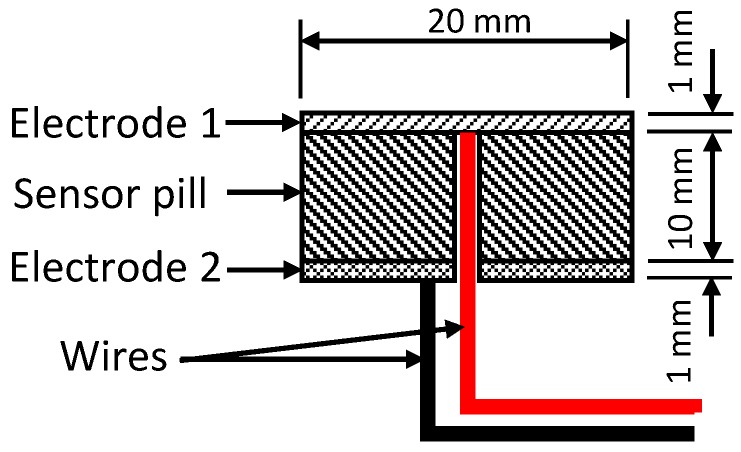
Electrode design of the sensor.

Transmission losses in the output signal and noise can be generated at the contact points between the sensor pills and electrodes. Two copper clad foils were used as electrodes while a layer of silver paste layer [39] is used in between the conductive polymer pill and the electrodes to minimise the above-mentioned phenomena (Figure 13). This novel wire piercing design method has significantly eased the routing provisions inside the sensor packaging, thus simplifying the packaging structure.

## 4. Results and Discussion

### 4.1. Characterisation of the Sensing Element

A core component of the tactile sensor is the conductive polymer based sensing element and the performance of the tactile sensor mostly depends on the characteristics of the sensing element. In order to find mechanical and material characteristics, a conductive polymer sample prepared with a weight ratio of 8% Super conductive carbon black to RTV Silicone Rubber (described by Huang et al. [38]) was used.

#### 4.1.1. Mechanical Characterisation of the Sensing Element

**Figure 14:**
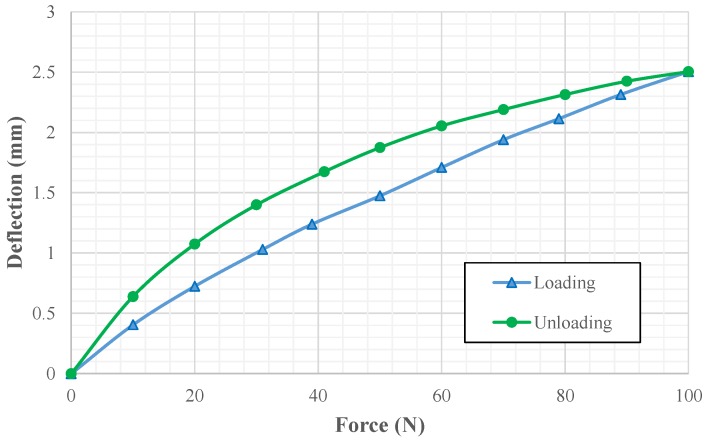
Experiment results: deflection vs. force for sensing element (only) using a force indenter.

The mechanical properties of the sensor pill was tested with respect to the deflection under loadings (Figure 14). A conductive polymer sample of 20 mm diameter and 10 mm thickness was tested with 10 N step loadings.

#### 4.1.2. Material Characterisation of the Sensing Element

**Figure 15:**
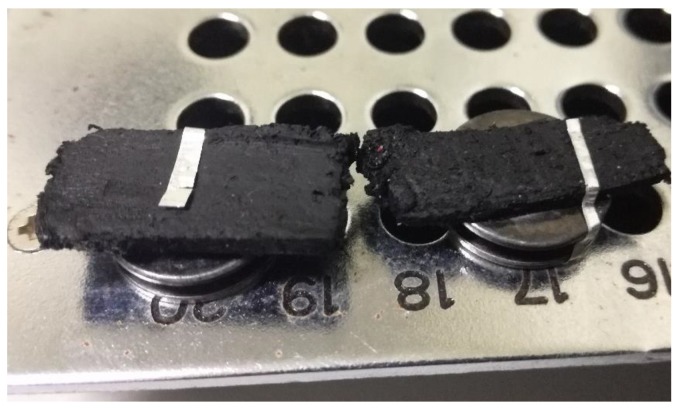
Conductive polymer samples prepared for microscopic analysis.

In order to conduct a microscopic and a particle analysis for the conductive polymer samples, a sample preparation method was required. First, the conductive polymer pill was sliced into samples each with a thickness of 2–3 mm using a precision low speed saw and a diamond blade. Later, a partial metallizing using gold sputtering was conducted prior to the analysis. Prepared samples that were mounted on Scanning Electron Microscope (SEM) studs are shown in Figure 15.

**Figure 16:**
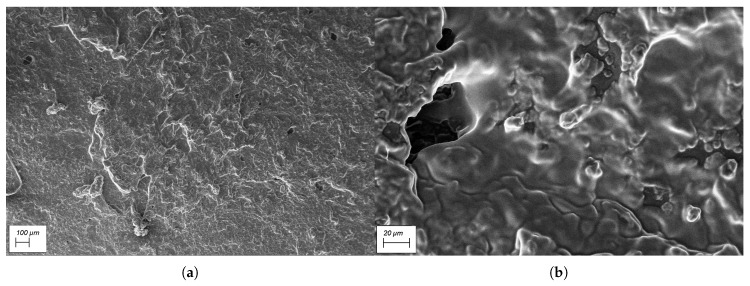
Microscopic images obtained using SEM: (**a**) at 100× magnification; (**b**) at 1000× magnification.

Microscopic images obtained using a Scanning Electrode Microscope (SEM) for the prepared conductive polymer samples are shown in Figure 16. It can be confirmed that the prepared samples were porous, even though they were prepared using a mechanical press moulding method.

**Figure 17:**
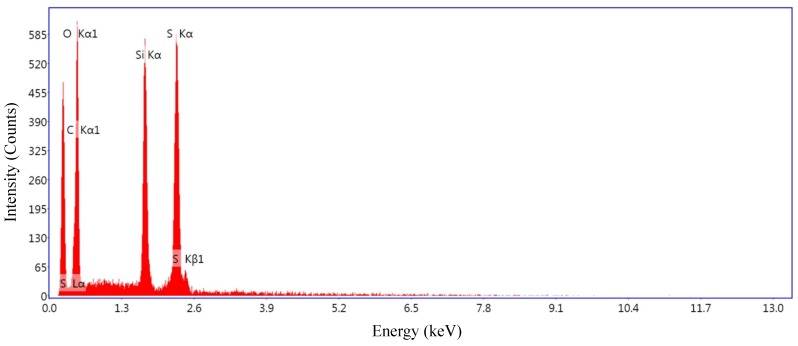
EDAX spectrum of the analysed sample.

The Energy-Dispersive X-ray (EDAX) spectrum for a prepared conductive polymer sample is illustrated in Figure 17. In the obtained elemental composition, only sulphur, oxygen, silicon and carbon signals can be observed. These have been identified as the major elements in the conductive samples prepared by Ying Huang et al [38] that was adopted for conductive polymer preparation. EDAX analysis shows the indirect evidence of a presence of nano-Silica (SiO${}_{2}$) that was used to enhance the conductivity of the polymer.

### 4.2. Signal Conditioning and Data Acquisition

**Figure 18:**
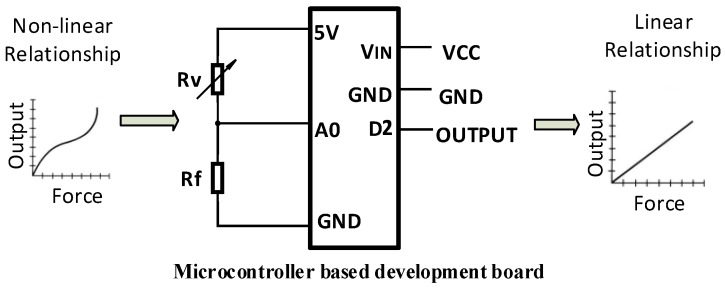
Developed electric circuit.

The developed electrical circuit design (Figure 18, $R_{v}$ denotes the sensing element) uses a micro controller based development board for further signal conditioning and controlling. A 10-bit Analog to Digital Converter(ADC) was utilised to obtain a voltage sensitivity of 0.0048 V. Deriving linear relation from nonlinear outputs taken from sensor pill considering loading condition and hysteresis errors was implemented in the system using a micro controller based development board. Look up table, based on experimental values would be used to obtain force sensor readings from the sensor which also mitigated the nonlinearity of the sensor.

### 4.3. Characterisation of the Sensor

**Figure 19:**
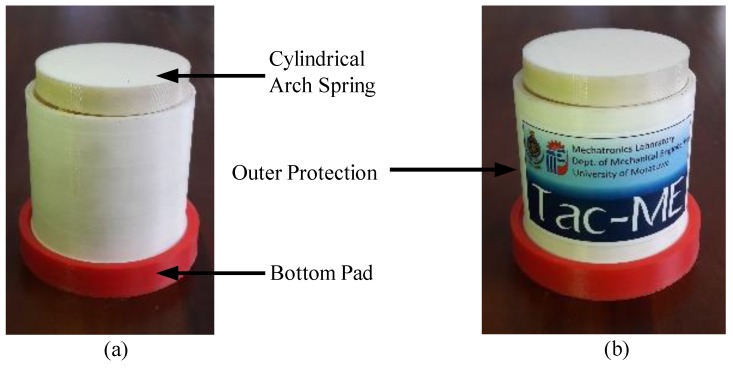
(**a**) Assembled sensor package; (**b**) “Tac-ME”: a 1-DoF Tactile sensor with the insertion of conductive polymer based sensing elements.

3D printed parts of the force sensor shown in Figure 10 and electrode embedded polymer pill were assembled Figure 19a. Assembled 1-Degree of Freedom (DoF) tactile sensor, “Tac-ME” (Figure 19b) was further tested to identify its mechanical behaviour and its performance.

**Figure 20:**
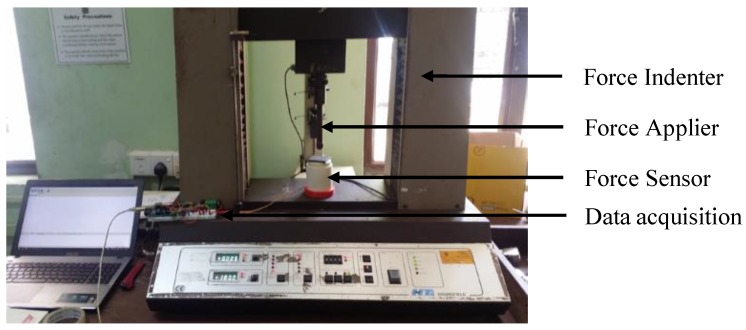
Force indenter is used for sensor characterisation.

**Figure 21:**
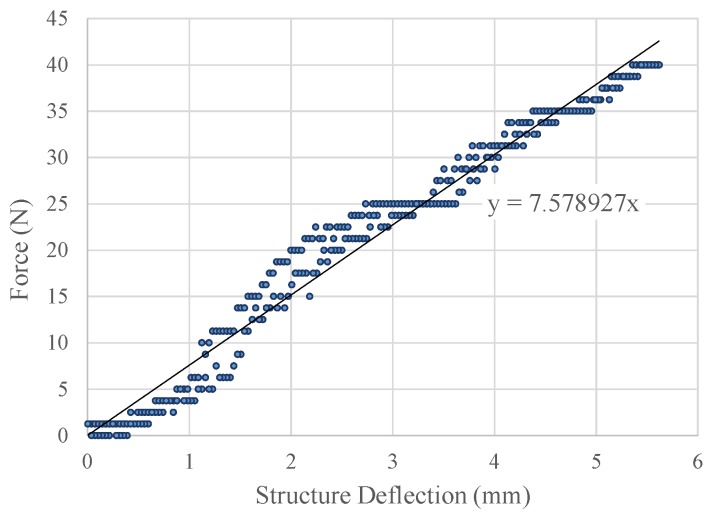
Experiment results: force vs. structure deflection for sensor structure (only) using the force indenter during one testing cycle.

At the beginning, the force indenter testing (Figure 20) for the entire spring structure (without the sensor pill insertion) gave the force vs. spring structure deflection graph as shown in Figure 21 for one testing cycle. The test was carried out as a ten cycle loading and unloading test. The maximum standard deviation was recorded as 0.117 mm (Table 2) for deflection for a given force.

The average stiffness for the structure was recorded as 7701.12 N/m with a standard deviation of 196.77 N/m. The experimental results for the stiffness had a deviation of 29.27% compared with the simulation results. The layering technique of 3D printing might be a major reason, whereas the 90${}^{\circ }$ diagonal material layering has negative and adverse concerns over the structural simulation assumption of solid and consistent ABSplus material in the sensor structure. Due to the fact of homogeneous characteristics of the structure being enhanced, the parallel material layering in 3D printing would diminish the deviations between the simulated results and the actual results.

Experiment results and performance of the sensor package, “Tac-ME” (Figure 19b) is obtained using a universal force indenter and dead-weights for the fabricated sensing structure and the sensing element respectively for their loading–unloading output characteristics.

**Figure 22:**
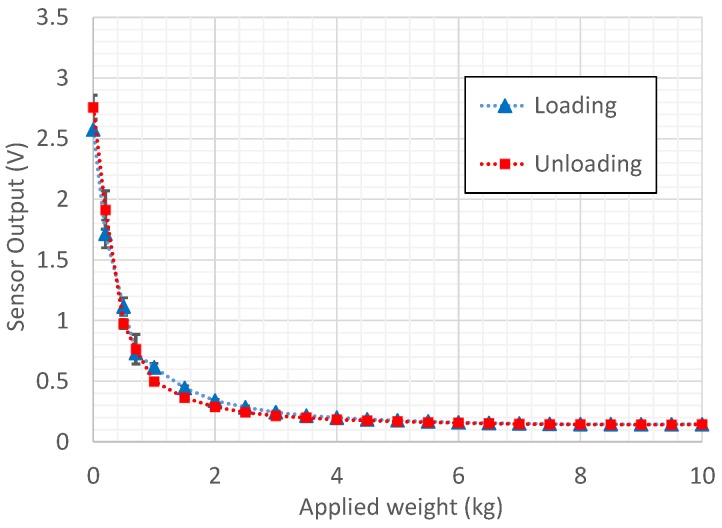
Experiment results: sensor output voltage vs. applied weight plot obtained using dead-weight testing with error bars based on standard deviation.

First, the packaged force sensor unit was tested under different methods to find out basic characteristics such as conductivity, force range, hysteresis and nonlinearity. The sensor output characteristics graph (with the variation of its standard deviation) in Figure 22 was obtained using four consecutive load tests using the force indenter. It hardly shows a hysteresis error and shows a significant repeatability due to small error bars that are present, which is a promising aspect in prospective applications. Force measurement using the developed sensor was carried out by comparing the voltage output of the sensor with look-up table values (based on Figure 22) obtained for each 10 N force interval.

**Figure 23:**
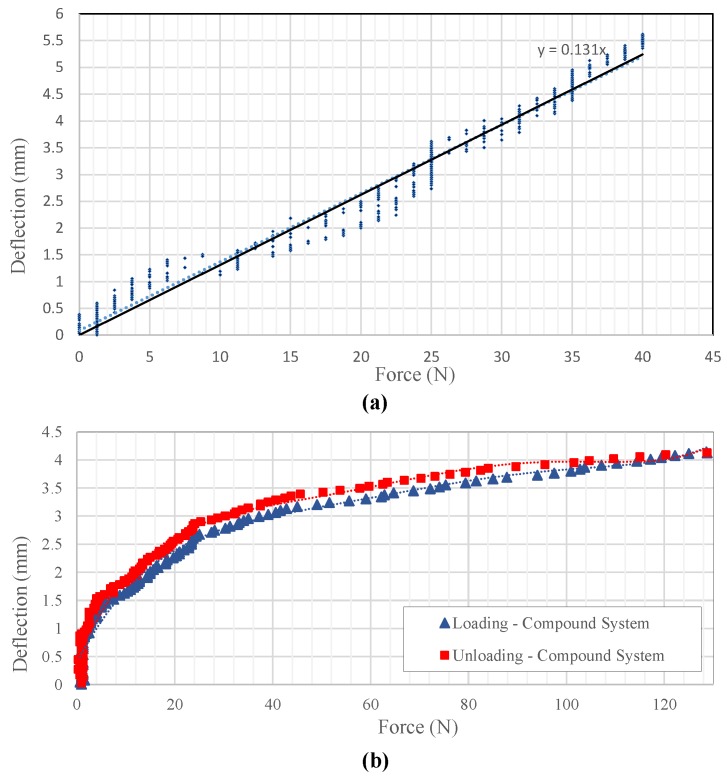
Experiment results using force indenter: deflection vs. applied force plots (**a**) structure only; (**b**) compound system.

The resultant plot of deflection vs. force applied using the force indenter only for the structure and for the compound system are shown under Figure 23, and its behavior highlighted particular amounts of mechanical hysteresis between its loading and unloading curves.

**Figure 24:**
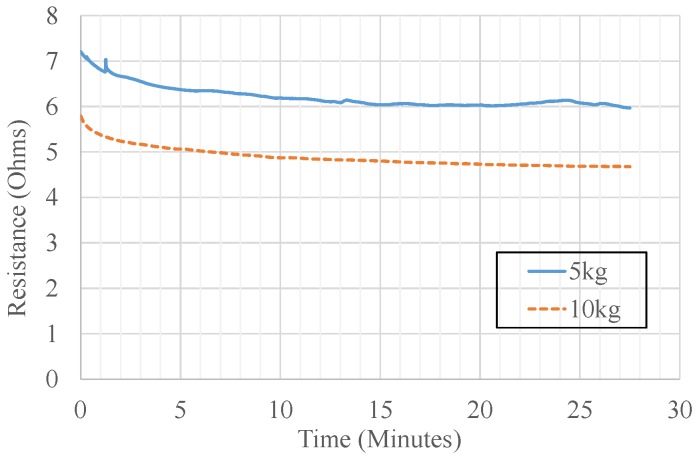
Experiment results: sensor drift plot—resistance change over the time.

Since the sensor response variation with respect to time is an essential aspect, an experiment was carried out to identify the sensor drift. The resistance was recorded using a high precision multi-meter. According to literature, the porosity of the conductive polymers causes the drift. The drift characteristics have been elaborated in Figure 24 where the output of the sensor, which successfully converges, was observed for two steady loadings (5 kg and 10 kg) for 27 min.

### 4.4. Communication

**Figure 25:**
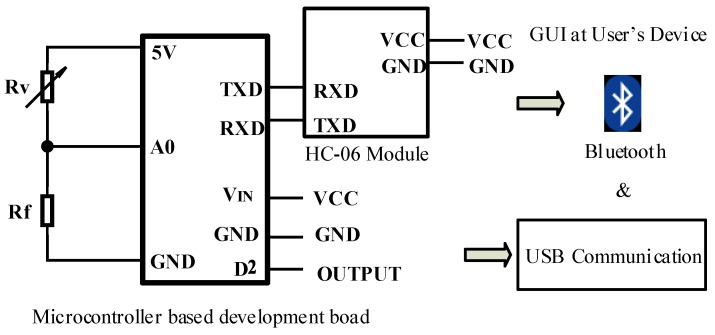
Circuitry for the developed communication modes.

The developed force sensor is capable of using two modes of communication to transmit sampled force sensing data to the user’s device. According to the pin diagram illustrated in Figure 25, the wireless communication via Bluetooth and the communication via Universal Serial Bus (USB) were the two modes used to communicate the said data. When using Bluetooth, the force sensor is automatically set to connect with the user’s device using the HC-06 Bluetooth modules attached to the micro-controller based development board and the Bluetooth connection available in the user’s device.

On the other hand, the sensing device can be connected to the users’ devices via USB connections. This allows the sensor to run on its internal power supply.

### 4.5. Discussion

Since the inception of this study, coming up with a novel tactile sensor unlike state-of-the-art macro level or micro level tactile sensors was the objective. Macro sized tactile sensors which have rigid sensing structures are mostly made out of bulk metal (e.g., —Aluminium) and use strain gauges as the sensing element. On the other hand, micro sized tactile sensors use semiconductor materials for the sensing structure and use many sensing principles (e.g., —capacitive, piezoresistive, etc.) for perception. In macro sized tactile sensors, adaptability to specific needs are challenging since the strain gauge bonding to the sensor structure is a major aspect that has to be safeguarded, despite the complexity and/or custom need of the sensor structure. Most of the miniature commercial tactile sensors are based on the Micro Electro Mechanical Systems (MEMS) technology and it involves several fabrication steps [40]. During such a fabrication process, mask designing and mask transferring can be identified as highly expensive key steps. Whenever a slight change is required for the sensor, the whole mask is required to be designed and fabricated again and it involves a high cost again.

Using this customisable 3D printed sensor fabrication approach, we could develop products and easily make changes to the product at a comparably low cost as only the designing/analysis part requires a modification. Utilizing additive manufacturing methods to such approaches gives that flexibility. Moreover, a flexible sensing element, which could be used along with a sensor structure, has to be utilised since strain gauges cannot be bonded to 3D printed tactile sensors due to the unsuitable surface texture. Henceforth, conductive polymer sensing elements were developed and utilised in this study.

## 5. Conclusions

A conductive polymer sensing element was used successfully in this novel sensor design by incorporating the piezoresistive behaviour of composites. The deflection of the sensing element can be controlled to vary within the required deflection range by using a customisable structure (fabricated using 3D printing technology) for the sensor. Due to the manufacturing techniques that have been followed, making different sizes and shapes of the sensing element is possible through press moulding and multiple force scaling structures can be 3D printed on a surface to fabricate sensor arrays.

A novel force scaling structure was designed, validated and developed successfully in this study. The design analysis methodology could be followed to design more sensors based on this design. However, customisability of the sensor requires specialized structural analysis. By the novel piercing wire routing method, packaging complexity has been reduced to a great extent. Sensor components’ assembling is achieved by threaded (3D printed) screw fixing, where the sensor packaging itself has overload protection.

The sensor possesses a sensitivity of 10 N with a range of 100 N. The sensor has a hysteresis of 21.7 Nmm per cycle and the repeatability is achieved with a 0.157 V maximum standard deviation. The sensing element itself has a drift as depicted in Figure 24. The implementation of the sensor with the wireless data acquisition capability, allows the sensor to be used in tactile sensor networks [41].

## Figures and Tables

**Table 1:** Different sensing element sizes.

Geometry	Diameter D (mm)	Thickness H (mm)
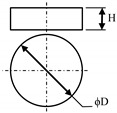	15	4, 7 and 10
	20	4, 7 and 10

**Table 2:** Force and structure deflection values for the sensor structure (only) during ten consecutive cycles.

Load (N)	Cycle 1 (mm)	Cycle 2 (mm)	Cycle 3 (mm)	Cycle 4 (mm)	Cycle 5 (mm)	Cycle 6 (mm)	Cycle 7 (mm)	Cycle 8 (mm)	Cycle 9 (mm)	Cycle 10 (mm)	Std. dev.
5	0.94	0.83	0.86	0.82	0.93	0.71	0.95	0.91	0.90	0.79	0.077
10	1.29	1.19	1.21	1.23	1.33	1.13	1.25	1.23	1.27	1.17	0.060
15	1.69	1.60	1.56	1.54	1.63	1.45	1.75	1.66	1.60	1.51	0.089
20	2.10	2.04	1.95	1.99	2.05	1.93	2.14	2.04	2.00	1.94	0.068
25	3.05	2.93	2.98	2.94	3.06	2.83	3.04	3.01	3.02	2.90	0.076
30	3.79	3.62	3.68	3.61	3.78	3.44	3.79	3.74	3.73	3.56	0.117
35	4.65	4.57	4.54	4.52	4.59	4.44	4.71	4.62	4.56	4.49	0.077
40	5.52	5.45	5.38	5.43	5.50	5.36	5.53	5.46	5.44	5.37	0.061

## Abbreviations

The following abbreviations are used in this manuscript:

ADC	Analogue to Digital Conversion
EDAX	Energy-Dispersive X-Ray
FEA	Finite Element Analysis
RTV	Room Temperature Vulcanization
SEM	Scanning Electron Microscope
USB	Universal Serial Bus
